# Longitudinal development of infant oral ecosystem: salivary metabolomic, bacteriome, and virome dynamics in early infancy

**DOI:** 10.1128/msystems.00946-26

**Published:** 2026-08-31

**Authors:** Ting Li, Xingyi Lu, Nora Alomeir, Anthony Gaca, Michael Sohn, Steven Gill, Bradley Smith, Joshua Munger, Jin Xiao

**Affiliations:** 1 Eastman Institute for Oral Health, University of Rochester Medical Center 6923, , Rochester, New York, USA; 2 Genomic Center, University of Rochester Medical Center 12299, Rochester, New York, USA; 3 Department of Biostatistics and Computational Biology, University of Rochester Medical Center 12299, Rochester, New York, USA; 4 Department of Microbiology and Immunology, University of Rochester Medical Center 12299, Rochester, New York, USA; 5 Department of Biochemistry and Biophysics, University of Rochester Medical Center 12299, Rochester, New York, USA; The Forsyth Institute, Cambridge, Massachusetts, USA

**Keywords:** bacteriome, virome, metabolome, early childhood, multi-omics

## Abstract

This prospective cohort study investigated the longitudinal development of the salivary bacteriome, virome, and metabolome during early infancy. We assessed the associations between oral bacteria, viruses, and metabolites from 10 mother-infant dyads, with oral samples collected at 1 and 2 years of age. Forty saliva and plaque samples underwent untargeted metabolomic analysis, and infant saliva samples underwent metagenomic sequencing. Maternal salivary and plaque metabolomic profiles remained largely stable, whereas infant profiles were clearly separated from maternal profiles and changed with age. Notably, infant dental plaque metabolism underwent more substantial changes from year 1 to year 2 than saliva, with age-dependent metabolite shifts mainly involving energy, amino acid, nucleotide, and lipid metabolic pathways. Our findings also revealed significant developmental shifts in salivary bacteriome, virome, and functional pathway profiles during early childhood. The most abundant oral bacteria in early life, comprising over 75% of total abundance, included *Veillonella, Streptococcus, Rothia, Prevotella, Neisseria*, and *Actinomyces species*. While human viruses like *Roseolovirus* were detected, bacteriophages constituted the majority of the virome. Comparing infants at year 1 and year 2, we identified differentially abundant bacteria, viruses, metabolic functional pathways, and specific metabolites. We observed associations between bacteria and viruses, noting that these cross-kingdom relationships attenuated as infants grew. The study results underscore the complex and dynamic development of the oral microbiome, virome, and metabolome during early childhood.

**IMPORTANCE:**

The human oral cavity undergoes substantial microbial and metabolic development during early childhood, yet the temporal changes in the infant oral ecosystem remain incompletely understood. In this study, we longitudinally profiled the salivary metabolome, bacteriome, and virome of infants at 1 and 2 years of age. We demonstrated that the infant oral metabolome undergoes substantial developmental shifts, particularly in pathways related to energy, amino acid, and lipid metabolism; whereas maternal metabolic profiles remained stable over the same period. Furthermore, our results revealed the dynamic assembly of infant salivary virome and bacteriome and their associations with the functional pathways and metabolites. These findings provide new insights into the complex and dynamic development of the oral microbiome, virome, and metabolome in early infancy.

## INTRODUCTION

Early infancy is a critical period for the development of oral and systemic health. The oral cavity, one of the most densely populated microbial habitats in the human body, is where microorganisms begin to colonize and grow within hours of birth (1). The development and maturation of the oral microbiome are significant because it plays a foundational role in both oral and systemic health, influencing immune system maturation, metabolic and neurological functions, and the risk of future oral diseases (2). Disturbances in this process, which can be triggered by host immune immaturity, environmental factors, antibiotic exposure, or diet, may have long-term effects on both oral and systemic health outcomes (3). Early microbial dysbiosis can negatively affect oral health in early childhood, mostly notably through the onset of dental caries, the most prevalent chronic illness in children (4). Early childhood caries causes pain, infection, delayed growth, and a decreased quality of life (5). In the United States, dental caries affects over 20% of children under 5, with the prevalence reaching approximately 40% among low-income and disadvantaged populations (6).

Metabolites provide a functional readout of oral ecosystem processes because they arise from host metabolism, oral microbial activity, and dietary intake (7). Saliva and dental plaque, however, represent complementary ecological compartments. Saliva is a rapidly renewed, well-mixed fluid that integrates host secretions and material shed from multiple oral surfaces, whereas supragingival plaque is a spatially organized, surface-associated biofilm in which microbial products and dietary substrates can accumulate. Metabolites such as lactate and acetate reflect carbohydrate fermentation and acidification, while amino acid and peptide derivatives may reflect tissue turnover, microbial metabolism, and host defense (8–12). Analyzing both matrices can, therefore, distinguish broadly distributed salivary signals from localized metabolic activity associated with biofilm maturation.

Untargeted metabolomics can provide an initial, matrix-resolved view of developmental remodeling before taxonomic and viral patterns are interpreted. Paired maternal samples offer a mature adult reference within the same family environment: stability across maternal visits can help distinguish infant developmental change from broader temporal variation, whereas mother-infant separation can indicate how far the developing oral metabolome remains from an adult state. Among oral matrices, saliva is particularly suitable for subsequent longitudinal multi-omics because it can be collected noninvasively and supports parallel metabolomic and metagenomic measurements. Nevertheless, plaque remains an important complementary matrix because its spatially organized biofilm can retain local substrates and microbial products.

Dental caries is a multifactorial disease, with host, microbial, and dietary factors, including sugar, contributing to its development (13). Most studies have focused on the bacterial community, identifying particular taxa associated with both healthy and diseased conditions (14). While other early colonizers, such as *Streptococcus salivarius, Streptococcus oralis, Veillonella* spp., and *Actinomyces* spp., play an essential role in the initial formation of biofilm communities, *Streptococcus mutans,* in particular, has long been identified as a culprit for dental caries (15). However, bacteria are just one aspect of the situation. The oral virome, which encompasses bacteriophages and eukaryotic viruses, is gaining increasing recognition as a significant modulator of microbial ecology (16). While bacteriophages can control bacterial population dynamics through predation and horizontal gene transfer, eukaryotic viruses, such as human herpesviruses, may have a direct impact on host immunity, epithelial barrier function, and oral bacteriome development (17, 18). As a result, these viral entities may act as keystone regulators in the development of the oral microbiome. However, their role during early childhood remains underexplored (19).

Accordingly, we used a staged analytical framework. We first applied untargeted metabolomics to paired mother-infant saliva and plaque to determine (i) whether maternal and infant profiles differed in longitudinal stability and global distribution and (ii) whether age-associated remodeling differed between saliva and plaque. Because maternal profiles were comparatively stable whereas infant profiles were distinct and developmentally dynamic, subsequent analyses focused on paired infant saliva to determine (iii) how the bacteriome, virome, and metagenomic functional potential changed from year 1 to year 2 and (iv) how salivary bacteria, viruses, and metabolites were associated at each age. We hypothesized that maternal metabolomes would remain relatively stable and that infant metabolomes would be clearly distinct and show greater longitudinal remodeling, particularly in plaque. We further hypothesized that the infant salivary bacteriome and virome would undergo dynamic developmental shifts with age and exhibit associations with oral metabolites.

## MATERIALS AND METHODS

### Study cohort

The study cohort consisted of 10 pregnant women who received dental care at the University of Rochester Eastman Institute for Oral Health Perinatal Dental Clinic during pregnancy and achieved a disease-free oral health status before delivery (7). The mothers and their infants were prospectively followed when infants were at 1 year and 2 years of age.

Mothers were excluded if they (i) had a decisional impairment, rendering them incapable of making an informed decision; (ii) received oral and/or systemic antifungal therapy within 90 days of the baseline study visit; (iii) used removable dental prosthesis for restoring missing teeth; (iv) had an orofacial deformity or tumor (e.g., cleft lip/palate); or (v) had a severe systemic disease (e.g., HIV infection).

Inclusion criteria for infants were as follows: (i) mothers were enrolled in the study; (ii) younger than 2 years of age at the initial visit. Infants were excluded if they had (i) had an orofacial deformity (cleft lip, cleft palate, or oral-pharyngeal mass), (ii) had a severe systemic disease, e.g., Down syndrome (7), or (iii) rhad eceived oral and/or systemic antifungal therapy within 90 days of the baseline study visit.

Enrollment began on 11 October 2022, and the final study visit was completed on 25 October 2023.

### Sample collection

Comprehensive oral examinations were conducted by one of the two calibrated study dentists at each study visit. Saliva and supragingival dental plaque samples were collected using rigorously standardized procedures to ensure data quality and reproducibility (8, 9). Participants were instructed to avoid eating, drinking, or brushing their teeth for 2 h before sample collection. For mothers, approximately 5 mL of unstimulated whole saliva was collected by having participants spit into a 50-mL sterile centrifuge tube. For infants, saliva was collected using a sterile swab, with infants chewing for 30–60 s. Supragingival plaque was collected from all teeth using a sterilized periodontal scaler and then suspended in 1 mL of sterile sodium chloride solution. Immediately after collection, both saliva and plaque samples were kept on ice and transported to the laboratory within 2 h. Oral samples were gently vortexed and sonicated in three cycles (each consisting of 10 s of sonication followed by 30 s on ice) to disperse any aggregates. The samples were then stored in the −80°C freezer.

### Metabolomics sample preparation

A volume of 100 µL of the saliva sample was extracted in 400 uL of 50:50 ACN:MEOH at −20°C for 30 min and kept then on regular ice for 30 min with vortexing every 10 min. Next, samples were centrifuged at 17,000 × *g* for 10 min, and 90% of the supernatant was dried down in a vacuum evaporator (Thermo). Samples were reconstituted in 50% acetonitrile (A955, Fisher Scientific), at a volume equal to 40% of dried-down volume and transferred to glass vials for LC/MS analysis.

Plaque samples were homogenized in 1 ml 40:40:20 ACN:MEOH:H₂O using a Precellys cold tissue homogenizer (Bertin), at a ratio of 36.4 μg (mom) or 12.8 μg (baby) to 1 mL of the solvent. Following homogenization, samples were transferred to −20°C for 30 min and then kept on regular ice for 30 min with vortexing every 10 min. Next, the samples were centrifuged at 17,000 × *g* for 10 min, and 90% of the supernatant was dried-down in a vacuum evaporator (Thermo). The samples were reconstituted in 50% acetonitrile (A955, Fisher Scientific) at a volume equal to 20% of dried-down volume and transferred to glass vials for LC/MS analysis. An aliquot of each was also taken to make pooled QCs that were similarly prepared.

### Liquid chromatography-tandem mass spectrometry (LC-MS/MS)

For LC-MS analysis, metabolite extracts were analyzed using high-resolution mass spectrometry with an Orbitrap Exploris 240 (Thermo) coupled to a Vanquish Flex liquid chromatography system (Thermo). Two microliters of samples was injected on a Waters XBridge XP BEH Amide column (150 mm length ×2.1 mm id, 2.5 μm particle size) maintained at 25°C, with a Waters XBridge XP VanGuard BEH Amide (5 mm × 2.1 mm id, 2.5 μm particle size) guard column. For positive mode acquisition, mobile phase A was 100% LC-MS grade H_2_O with 10 mM ammonium formate and 0.125% formic acid. Mobile phase B was 90% acetonitrile with 10 mM ammonium formate and 0.125% formic acid. For negative mode acquisition, mobile phase A was 100% LC-MS grade H_2_O with 10 mM ammonium acetate, 0.1% ammonium hydroxide, and 0.1% medronic acid (Agilent). Mobile phase B was 90% acetonitrile with 10 mM ammonium acetate, 0.1% ammonium hydroxide, and 0.1% medronic acid. The gradient was 0 min, 100% B; 2 min, 100% B; 3 minutes, 90% B; 5 minutes, 90% B; 6 minutes, 85% B; 7 minutes, 85% B; 8 minutes, 75% B; 9 minutes, 75% B; 10 minutes, 55% B; 12 minutes, 55% B; 13 minutes, 35%; 20 minutes, 35% B; 20.1 minutes, 35% B; 20.6 minutes, 100% B; 22.2 minutes, 100% B; all at a flow rate of 150 μL/min, followed by 22.7 minutes, 100% B; 27.9 minutes, 100% B at a flow rate of 300 μL/min, and finally 28 minutes, 100% B at flow rate of 150 μL/min, for a total length of 28 minutes. The H-ESI source was operated in positive mode at spray voltage 3,500 or negative mode at spray voltage 2,500 with the following parameters: sheath gas 35 au, aux gas seven au, sweep gas 0 au, ion transfer tube temperature 320°C, vaporizer temperature 275°C, mass range 70 to 1,000 *m*/*z*, full scan MS1 mass resolution of 120,000 FWHM, RF lens at 70%, and standard automatic gain control (AGC). LC-MS data were analyzed by Compound Discoverer (v3.3, Thermo Scientific) and El-Maven software (reference below) for peak area determination and compound identification. Compounds were identified by matching to LC-MS method-specific retention time values of external standards and MS2 spectral matching to external standards and the mzCloud database (Thermo Scientific).

### DNA extraction, NGS library construction, and sequencing

Total genomic DNA was extracted from the oral samples using a modification of the ZymoBIOMICSTM DNA Miniprep Kit (Zymo Research, Irvine, CA) and FastPrep mechanical lysis (MPBio, Solon, OH). The quality of extracted genomic DNA (gDNA) was assessed on a Fragment Analyzer (Agilent Technologies, Inc.) and quantified using Qubit (ThermoFisher Scientific). Libraries were constructed using the Nextera XT kit (Illumina, Inc.) with up to 1 ng of gDNA as input. Fragment size profiles and quantification of the libraries were measured using the Fragment Analyzer and Qubit, respectively. The libraries were normalized to 2 nM, pooled at equimolar concentrations, and sequenced on NovaSeq 6000 with 150 cycle paired-end reads. Raw reads generated from the Illumina basecalls were demultiplexed using BCL Convert version 4.1.7. Illumina adapters, prokaryotic rRNA, and host reads were removed from the raw sequencing reads using KneadData (v0.7.4). The filtered read files underwent bacterial taxonomic and functional classification using MetaPhlAn (v3.0.5) and HUMAnN (v3.0.1). Viral taxa were quantified using Kraken2 (v2.1.3) and the pre-built viral RefSeq database. Bracken (v2.9) was used to re-estimate species-level and genus-level abundances.

### statistical analysis

Data from the raw metabolomics outputs were processed using Thermo Scientific’s Compound Discoverer through a curated database search. The raw area counts of each metabolite peak were normalized to pooled QC samples run throughout the LC/MS run. Metabolites with *P*-value <  0.05 after the False Discovery Rate (FDR) correction were characterized as statistically significant. Principal component analysis (PCA) was conducted on the auto-scaled data to assess global metabolic variation and sample clustering. Differential metabolites were visualized using volcano plots, plotting log₂ fold change against −log_10_ (*P*-values). Pathway analysis was performed using MetaboAnalyst 6.0 with significantly altered metabolites as input. The hypergeometric test was used for pathway enrichment, relative betweenness centrality for topology analysis, and Homo sapiens (KEGG) as the reference library. Pathway networks were visualized using Cytoscape 3.10.3.

All statistical tests were conducted as two-sided with a significance level of α = 0.05. Statistical analyses were performed using R v4.4.1 (R Foundation for Statistical Computing, Vienna, Australia). Data visualization was conducted using GraphPad Prism 9.5.0 (GraphPad Software, Boston, MA). Prior to downstream analysis, metagenomic sequencing reads were converted into compositional data.

For alpha diversity analyses, the Shannon diversity index was calculated for bacteriome, virome, and functional pathways, without filtering criteria for detected taxa and pathways. Comparisons of alpha diversity between year 1 and year 2 samples were performed using the mixed-effects model. Beta diversity was assessed using the Bray-Curtis dissimilarity index to measure differences between sample pairs, without filtering criteria for detected taxa and pathways. Permutational multivariate analysis of variance (PERMANOVA) was used to compare beta diversity between year 1 and year 2 samples.

To identify differential abundant bacteria, viruses, and functional pathways between years 1 and 2, we used a criterion that each bacterial, viral, or pathway feature must be present in at least two samples per group with a prevalence filtering criterion of greater than 20% among the samples of each time point. We then utilized the method OPTIMEM (an optimal normalization method for high sparse compositional data) (20). The bacteriome, virome, and functional pathway reads were first converted to compositional terms. OPTIMEM then generated a set of non-differentially abundant taxa, and the abundance of all taxa and pathways was divided by the sum of the abundances of this non-DA set, followed by a logarithmic transformation. Subsequently, a mixed-effect model was performed, and *P*-values were adjusted using the Benjamini-Hochberg method.

We investigated correlations between bacteria and viruses, bacteria and metabolites, and viruses and metabolites. To account for compositional effects, these correlations were analyzed using binary data (presence and absence) rather than continuous abundance data across year 1 and year 2. Pearson’s correlation analysis was performed, and *P*-values were corrected for multiple testing using the Benjamini-Hochberg method. For display purposes, at each time point, only variables with at least one significantly correlated variable were presented. For instance, in the bacteria-virus correlation heatmap, only bacteria with one or more significantly correlated viruses were displayed and vice versa. To illustrate the connections between functional pathways and the salivary microbiome, Cytoscape was used for visualization. Information regarding pathways was collected from MetaCyc, and differentially abundant pathways were distinctly marked with larger fonts and more visible colors to highlight their significance.

## RESULTS

### Untargeted metabolomics reveals stable maternal profiles and marked infant-maternal divergence

**Fig 1:**
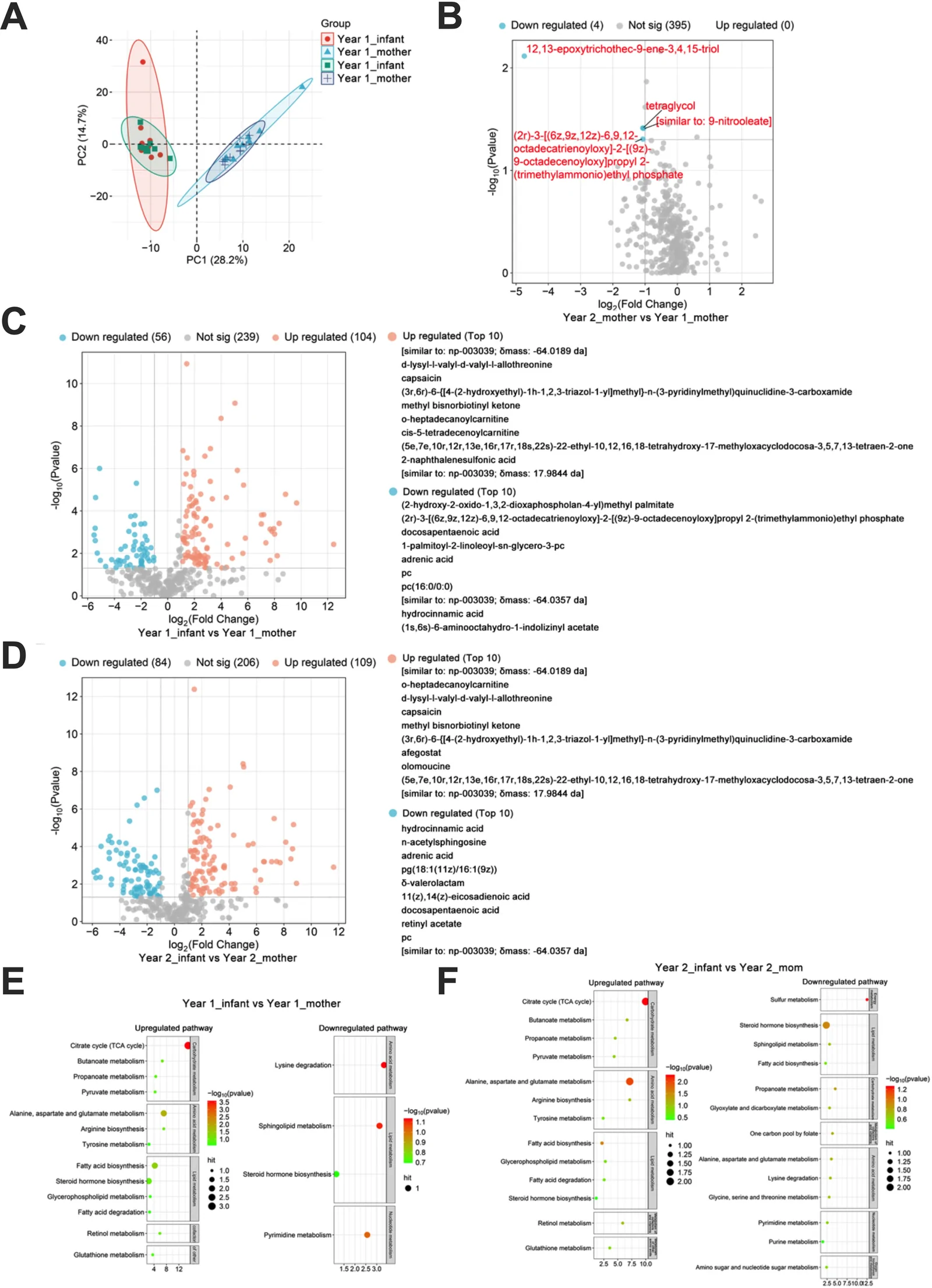
Saliva metabolomic differences between infants and mothers. (**A**) PCA score plot of untargeted metabolomics data for saliva; each dot represents a biological sample. (**B**) Volcano plot showing differential metabolites in year 2 versus year 1 maternal saliva, with four downregulated metabolites (*P* < 0.05, log₂FC < −1). (**C**) Volcano plot of differential metabolites in year 1 infant versus year 1 maternal saliva, showing 104 upregulated (*P* < 0.05, log₂FC > 1) and 56 downregulated (*P* < 0.05, log₂FC < −1) metabolites. (**D**) Volcano plot of differential metabolites in year 2 infant versus year 2 maternal saliva, showing 109 upregulated (*P* < 0.05, log₂FC > 1) and 84 downregulated (*P* < 0.05, log₂FC < −1) metabolites. (**E**) Pathway enrichment analysis of up- and downregulated metabolites in year 1 saliva based on human metabolic pathways. (**F**) Pathway enrichment analysis of up- and downregulated metabolites in year 2 saliva based on human metabolic pathways.

Untargeted metabolomics first established the developmental contrast between mothers and infants. Principal component analysis (PCA) of saliva showed pronounced separation of maternal and infant samples at both visits, while maternal samples clustered closely across time (Fig. 1A). Only four maternal salivary metabolites decreased from year 1 to year 2 (Fig. 1B). In contrast, 160 metabolites differed between infants and mothers at year 1 and 193 at year 2 (Fig. 1C and D). Infant-enriched features mapped to the TCA cycle, amino acid metabolism, and fatty acid biosynthesis, whereas infant-depleted features mapped to steroid hormone and nucleotide metabolism (Fig. 1E and F).

Plaque showed the same developmental pattern. Maternal plaque profiles overlapped substantially between visits, with only one metabolite increasing at year 2 (Fig. S1A and B), whereas infant and maternal plaque samples were widely separated and differed by 210 metabolites at year 1 and 220 at year 2 (Fig. S1C and D). Infant-enriched pathways included central carbon and amino acid metabolism at year 1, with additional lipid-related enrichment at year 2 (Fig. S1E and F).

Notably, pathway-level analysis revealed opposing trends among metabolites within the same pathways. For example, within steroid hormone biosynthesis, cholesterol sulfate consistently decreased while estrone glucuronide increased. Similar bidirectional regulation was observed in purine metabolism, glycine/serine/threonine metabolism, fatty acid biosynthesis, propanoate metabolism, and alanine/aspartate/glutamate metabolism (Fig. S2 to S7), indicating that pathway enrichment should not be interpreted as uniform activation or suppression of every constituent metabolite.

### Longitudinal metabolomic remodeling is greater in infant plaque than saliva

**Fig 2:**
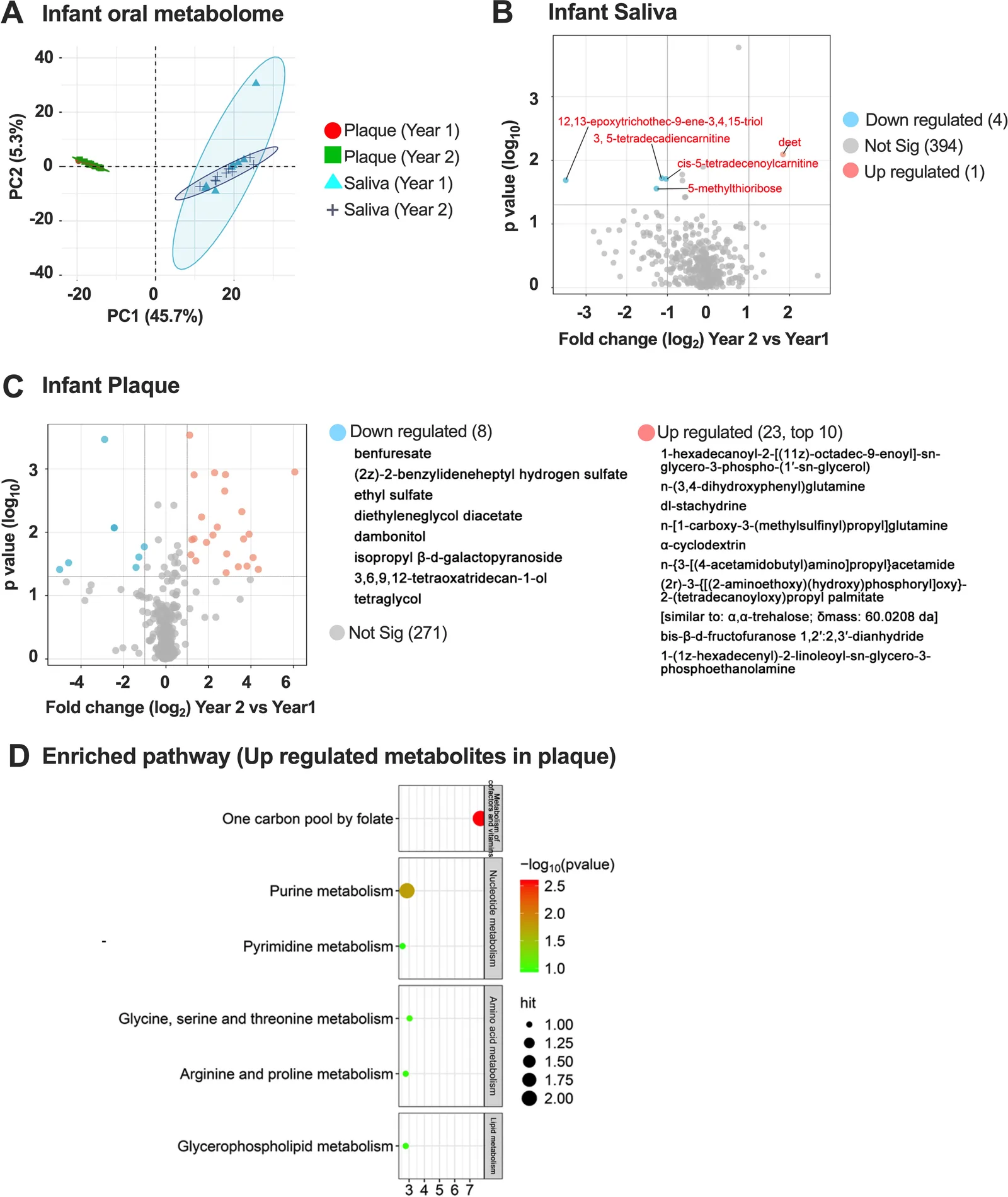
Metabolomic profiles and differential metabolites in infant saliva and plaque. (**A**) Two-dimensional principal component analysis (PCA) score plot of untargeted metabolomics data, with each point representing an individual sample. (**B**) Volcano plot of differential metabolites in infant saliva at year 2 versus year 1, showing one upregulated metabolite (*P* < 0.05, log₂FC > 1) and four downregulated metabolites (*P* < 0.05, log₂FC < −1). (**C**) Volcano plot of differential metabolites in year 2 versus year 1 infant plaque, showing 23 upregulated (*P* < 0.05, log₂FC > 1) and eight downregulated metabolites (*P* < 0.05, log₂FC < −1). (**D**) Pathway enrichment analysis of upregulated metabolites in infant plaque samples.

PCA confirmed data reproducibility and showed clear separation between saliva and plaque but relative stability across years within each sample type (Fig. 2A). Differential analysis identified five significant metabolites in infant saliva and 31 in plaque (Fig. 2B and C; Table 1).

**TABLE 1:** Age-associated oral metabolic shifts in 2-year-old infants

Metabolite	Origin	Pathway (based on origin)	Log2 FC	*P*-value	Interpretation
Saliva					
12,13-Epoxytrichothec-9-ene-3,4,15-triol	Fungal/mycotoxin	Fungal secondary metabolite biosynthesis	−3.498	0.021	Indicates less environmental fungal/toxin exposure or better oral barrier/microbial exclusion
5-Methylthioribose	Endogenous and microbial	Methionine salvage pathway/sulfur amino acid metabolism	−1.271	0.028	Suggests reduced microbial sulfur metabolism or tighter methionine recycling in older infants
3, 5-Tetradecadiencarnitine	Endogenous	Fatty acid β-oxidation/acylcarnitine metabolism	−1.141	0.019	Suggests less immune-metabolic stress or more stable oral energy homeostasis
*cis*-5-Tetradecenoylcarnitine	Endogenous	Fatty acid β-oxidation/acylcarnitine metabolism	−1.034	0.020	May reflect more efficient mitochondrial β-oxidation or reduced mucosal stress
Deet	Environmental/exogenous	Not endogenous	1.838	0.008	Likely due to increased environmental exposure or behavior-related contact (e.g., crawling, repellents, and toys)
Plaque					
Benfuresate	Environmental/exogenous	Xenobiotic metabolism	−5.006	0.038	Suggests reduced herbicide/xenobiotic exposure in older infants, possibly reflecting dietary/environmental changes
(2z)-2-Benzylideneheptyl hydrogen sulfate	Environmental/exogenous	Xenobiotic metabolism	−4.586	0.030	Likely indicates lower accumulation of environmental chemical residues in oral biofilms with age
Ethyl sulfate	Endogenous	Ethanol biotransformation	−2.887	0.000	Suggests lower microbial ethanol fermentation in plaque, consistent with oral microbiome maturation
Diethyleneglycol diacetate	Environmental/exogenous	Not endogenous	−2.436	0.009	Reflects reduced exposure to synthetic compounds (plasticizers/solvent residues), pointing to environmental factor reduction with age
Dambonitol	Plant polyol	Carbohydrate metabolism	−2.436	0.009	Reflects dietary shifts (e.g., reduced plant polysaccharide breakdown in plaque biofilm)
Isopropyl β-d-galactopyranoside	Environmental/exogenous	Not endogenous	−1.409	0.036	Indicates less exposure to synthetic sugars, consistent with age-related diet/feeding change
3,6,9,12-Tetraoxatridecan-1-ol	Environmental/exogenous	Not endogenous	−1.275	0.025	Suggests reduced accumulation of surfactant-like exogenous compounds in oral biofilms
Tetraglycol	Environmental/exogenous	Not endogenous	−1.020	0.017	Points to lower retention of exogenous glycol-type residues in plaque
n8-Acetylspermidine	Endogenous	Polyamine metabolism	1.114	0.000	Reflects enhanced polyamine turnover in maturing biofilm; linked to cell proliferation and immune modulation in the oral microenvironment
Vigabatrin	Synthetic drug	Drug metabolism and GABA-T inhibition	1.174	0.023	Indicates trace xenobiotic exposure or analytical overlap; unlikely endogenous but suggests higher environmental/drug-derived presence
Demethylalangiside	Plant (iridoid glycoside)	Plant secondary metabolism	1.221	0.013	Reflects greater exposure to dietary plant glycosides, consistent with solid food introduction in older infants
2-(Hydroxymethyl)-1-methylpiperidine-3,4,5-triol	Plant alkaloid (DNJ-like)	Sugar-alkaloid metabolism	1.325	0.013	Suggests microbial processing of plant-derived alkaloids; reflects a more complex oral-dietary interaction
Agmatine	Endogenous and microbial	Amino acid metabolism	1.325	0.001	Indicates enhanced microbial and host amino acid decarboxylation activity; may support biofilm growth and immune signaling
PC	Endogenous/dietary	Kennedy pathway and glycerophospholipids	1.406	0.028	Reflects increasing membrane turnover and glycerophospholipid metabolism in maturing plaque biofilm
Hypoxanthine	Endogenous	Purine metabolism	1.679	0.006	Suggests heightened purine turnover, possibly linked to microbial proliferation or host cell turnover in plaque
Daminozide	Agrochemical	Hydrolysis → succinate + dimethylhydrazine	1.907	0.014	Suggests increased agrochemical/xenobiotic intake (diet/environment-related)
1-Palmitoyl-2-linoleoyl-sn-glycero-3-pc	Endogenous	Glycerophospholipid metabolism	2.213	0.011	Indicates upregulated host or microbial phosphatidylcholine metabolism and membrane remodeling in plaque
Adenosine	Endogenous/dietary	Purine metabolism	2.289	0.001	Reflects heightened purine metabolism and potential immunoregulatory signaling in the plaque microenvironment
Choline	Endogenous/dietary	Choline metabolism (acetylcholine, betaine, and PC synthesis)	2.419	0.008	Consistent with enhanced phospholipid turnover, acetylcholine precursors, and microbial choline utilization
(15r,21s)-18,21,22-Trihydroxy-3-methyl-18-oxido-12-oxo-13,17,19-trioxa-18λ~5-phosphadocosan-15-yl 14-methylhexadecanoate	Endogenous	Glycerophospholipid metabolism	2.769	0.002	Reflects complex glycerophospholipid remodeling in biofilm/host membranes
[Similar to 4-dodecylbenzenesulfonic acid; δmass: −0.1191 Da]	Environmental/exogenous	Environmental contaminant	2.803	0.001	Indicates higher environmental contaminant accumulation with age (possibly detergents and plastics)
1-(1z-Hexadecenyl)-2-linoleoyl-sn-glycero-3-phosphoethanolamine	Host/microbes	Glycerophospholipid metabolism	2.836	0.044	Reflects active microbial/host glycerophospholipid remodeling in plaque
Bis-β-d-fructofuranose 1,2′:2,3′-dianhydride	Microbial/*Arthrobacter*	Carbohydrate metabolism	2.863	0.022	Suggests unique microbial carbohydrate metabolism (Arthrobacter-linked), possibly reflecting microbiome diversification
(2r)-3-{[(2-Aminoethoxy)(hydroxy)phosphoryl]oxy}-2-(tetradecanoyloxy)propyl palmitate	Host/microbes	Glycerophospholipid metabolism	3.574	0.005	Indicates complex glycerophospholipid biosynthesis in plaque (microbe-host interaction)
n-{3-[(4-Acetamidobutyl)amino]propyl}acetamide	Host	Polyamine metabolism	3.654	0.013	Indicates active polyamine metabolism, linked to microbial growth and oral mucosal interaction
n-[1-Carboxy-3-(methylsulfinyl)propyl]glutamine	Dietary/plant-derived/host	Amino acid or glutamine conjugates	3.913	0.011	Suggests introduction of sulfur-containing foods/metabolites with age
dl-Stachydrine	Dietary/plant-derived	Marker of citrus intake	4.103	0.025	Consistent with citrus/plant intake from diet expansion
n-(3,4-Dihydroxyphenyl)glutamine	Dietary/host	Aromatic amino acid/polyphenol catabolism	4.351	0.038	Indicates increased polyphenol metabolism (diet + microbial catabolism)
1-Hexadecanoyl-2-[(11z)-octadec-9-enoyl]-sn-glycero-3-phospho-(1′-sn-glycerol)	Host/microbes	Aromatic amino acid/polyphenol catabolism	6.068	0.001	Suggests active membrane lipid remodeling in maturing oral plaque biofilm

In saliva, year 2 infants showed reduced levels of a fungal toxin derivative, sulfur amino acid metabolites, and beta-oxidation intermediates, but higher DEET, likely reflecting increased environmental exposure. Plaque exhibited more extensive remodeling: environmental xenobiotics and microbial fermentation products decreased, whereas polyamines, purine metabolites, glycerophospholipids, and plant-derived compounds increased, indicating biofilm maturation and dietary diversification. Pathway analysis revealed enrichment in one-carbon pool by folate and purine metabolism, both essential for nucleotide biosynthesis and microbial proliferation (Fig. 2D).

### Age-associated changes in the infant salivary bacteriome and virome

**Fig 3:**
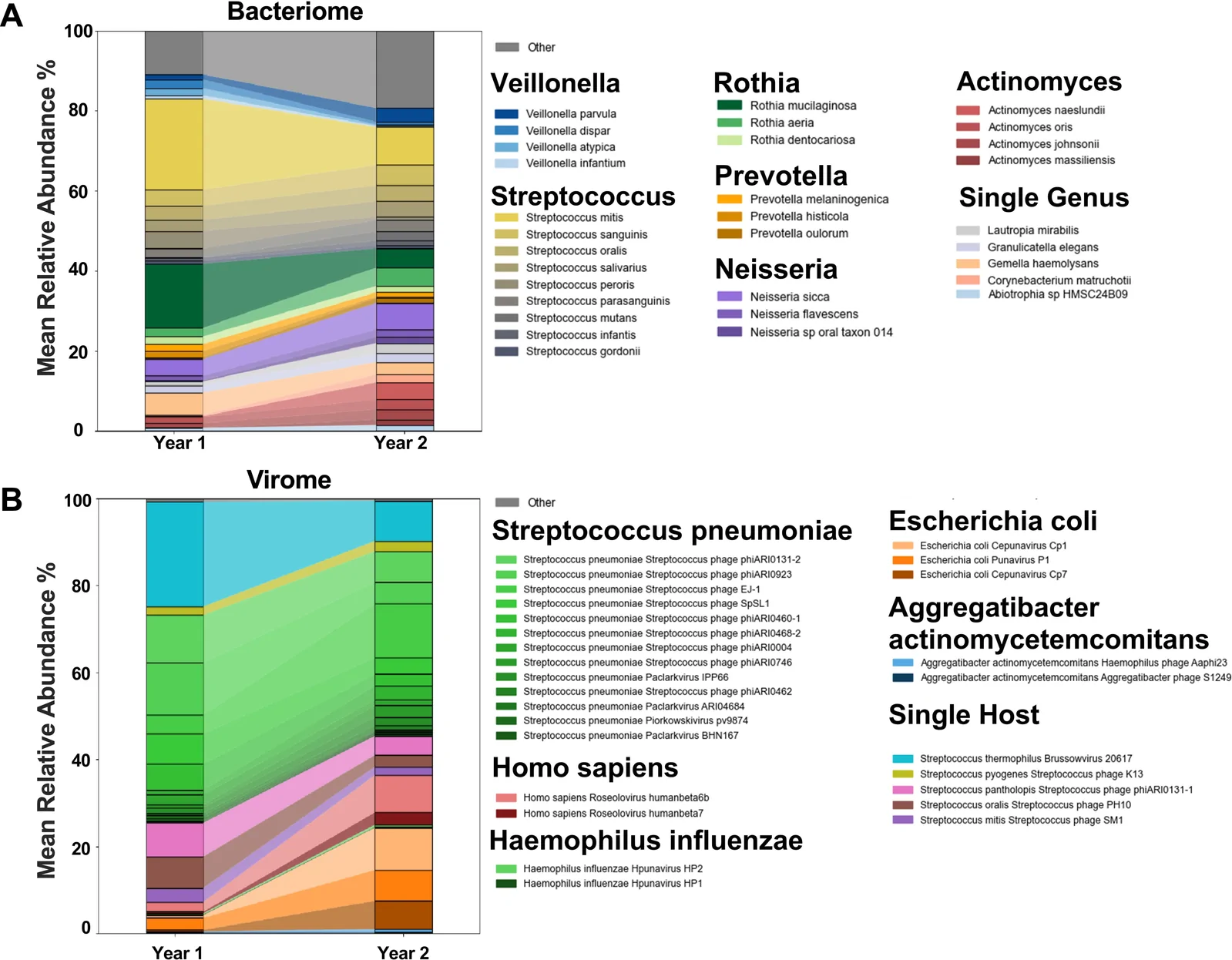
Bacteriome and virome profiles of infant saliva. (**A**) Relative abundances of the top 25 most abundant bacterial taxa in infant saliva at 1 and 2 years of age. (**B**) Relative abundances of the top 25 most abundant viral taxa in infant saliva at 1 and 2 years of age.

In total, we detected 283 bacterial taxa, 142 viral taxa, 365 metabolic pathways, and 399 metabolites in infant saliva samples. Distinct temporal patterns were observed in both salivary microbial and viral communities during the first 2 years of life. Within the bacteriome (Fig. 3A), several taxa exhibited marked shifts in relative abundance between year 1 and year 2. The 25 most abundant bacterial taxa collectively accounted for approximately 80%–85% of the total community. Specifically, *Veillonella parvula* demonstrated a significant increase in abundance from year 1 to year 2, while other *Veillonella* species generally showed decreased abundances. Various *Streptococcus* species, including *Streptococcus mitis*, *Streptococcus sanguinis, Streptococcus oralis, and Streptococcus salivarius*, typically decreased in abundance, with the most pronounced reduction observed for *Streptococcus mitis*. In contrast, *Actinomyces* species (*A. naeslundii, A. oris, A. johnsonii,* and *A. massiliensis*) exhibited a notable increase in abundance from year 1 to year 2. *Rothia* species (*R. mucilaginosa, R. aeria,* and *R. dentocariosa*) exhibited divergent trajectories; the abundance of *R. mucilaginosa* decreased from over 20% to less than 10%, whereas that of *R. aeria* increased from over 2% to over 4%.

The salivary virome also exhibited dynamic changes across time (Fig. 3B). The 25 most abundant viral taxa collectively accounted for over 100% of the total detected viral abundance. Notably, phages targeting *Streptococcus pneumoniae* maintained consistent abundances across both years. Additionally, phages infecting *Homo sapiens* (*Roseolovirus humanbeta6b* and *Roseolovirus humanbeta7*) and phages infecting *Escherichia coli* (*Cepunavirus Cp1, Cepunavirus Cp7,* and *Punavirus P1*) showed a significant increase in abundance from year 1 to year 2.

### Diversity of bacteriome, virome, and functional pathways

**Fig 4:**
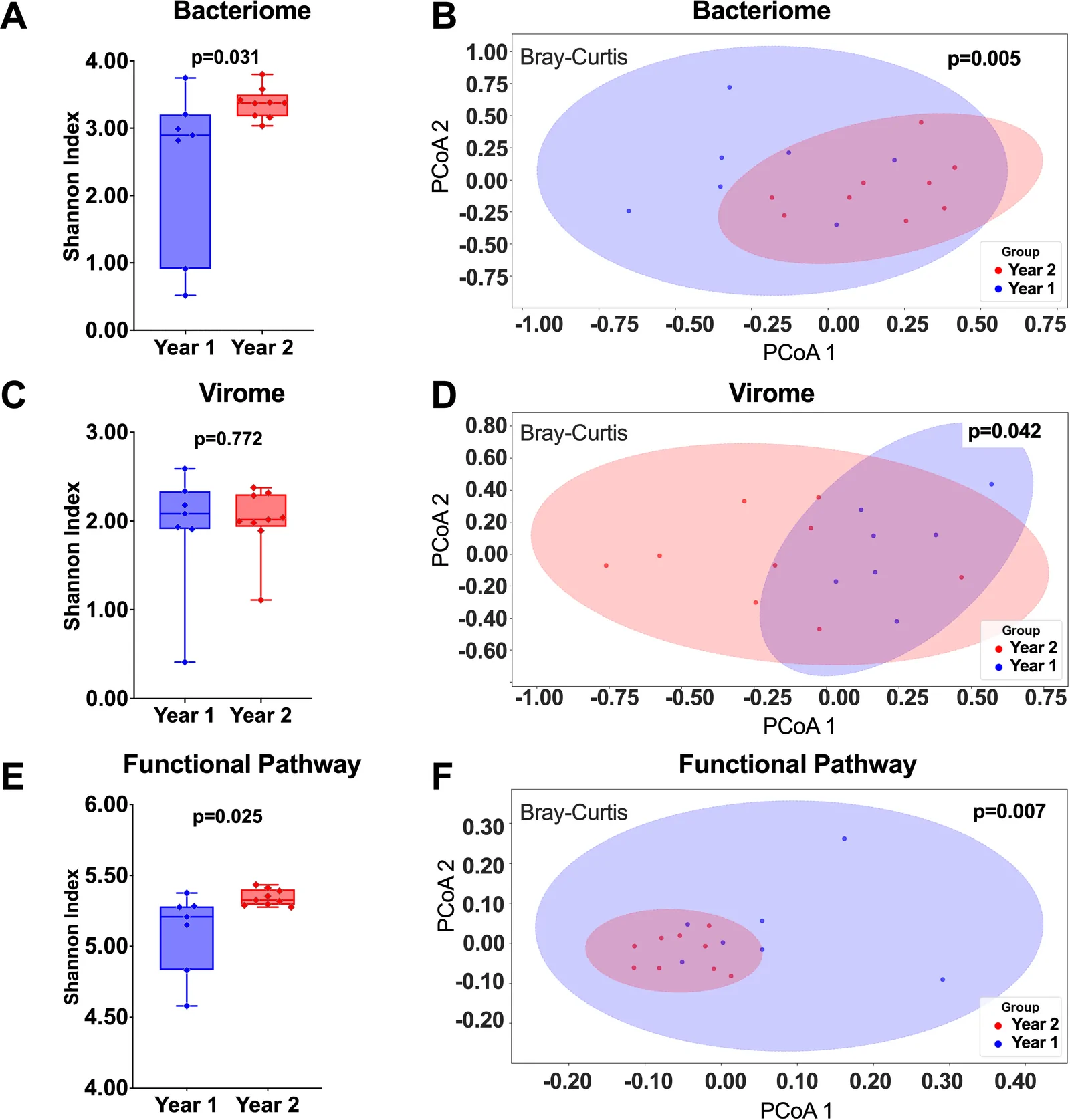
Diversity of the salivary bacteriome, virome, and functional pathways in early infancy. (**A**) Alpha diversity of the salivary bacteriome measured by the Shannon index. (**B**) Beta diversity of the salivary bacteriome assessed using the Bray-Curtis dissimilarity index. (**C**) Alpha diversity of the salivary virome measured by the Shannon index. (**D**) Beta diversity of the salivary virome assessed using the Bray-Curtis dissimilarity index. (**E**) Alpha diversity of salivary functional pathways measured by the Shannon index. (**F**) Beta diversity of salivary functional pathways assessed using the Bray-Curtis dissimilarity index. The mixed-effect model was used to compare alpha diversity between year 1 and year 2. PERMANOVA was used to test differences in beta diversity between time points.

Significant temporal changes were observed in microbial diversity metrics. Both alpha and beta diversity of the bacteriome differed significantly between year 1 and year 2 (Fig. 4A, *P* = 0.031 for alpha diversity; Fig. 4B, *P* = 0.005 for beta diversity). In contrast, virome alpha diversity remained stable over time (Fig. 4C, *P* = 0.772), whereas beta diversity differed significantly between the two time points (Fig. 4D, *P* = 0.042), indicating compositional restructuring without changes in overall richness or evenness. Functional pathway diversity also changed with age. Both alpha and beta diversity of salivary metabolic functional pathways showed significant differences between year 1 and year 2 (Fig. 4E, *P* = 0.025 for alpha diversity; Fig. 4F, *P* = 0.007 for beta diversity), suggesting developmental shifts in functional potential.

### Differential abundant bacteria, viruses, and functional pathways

**Fig 5:**
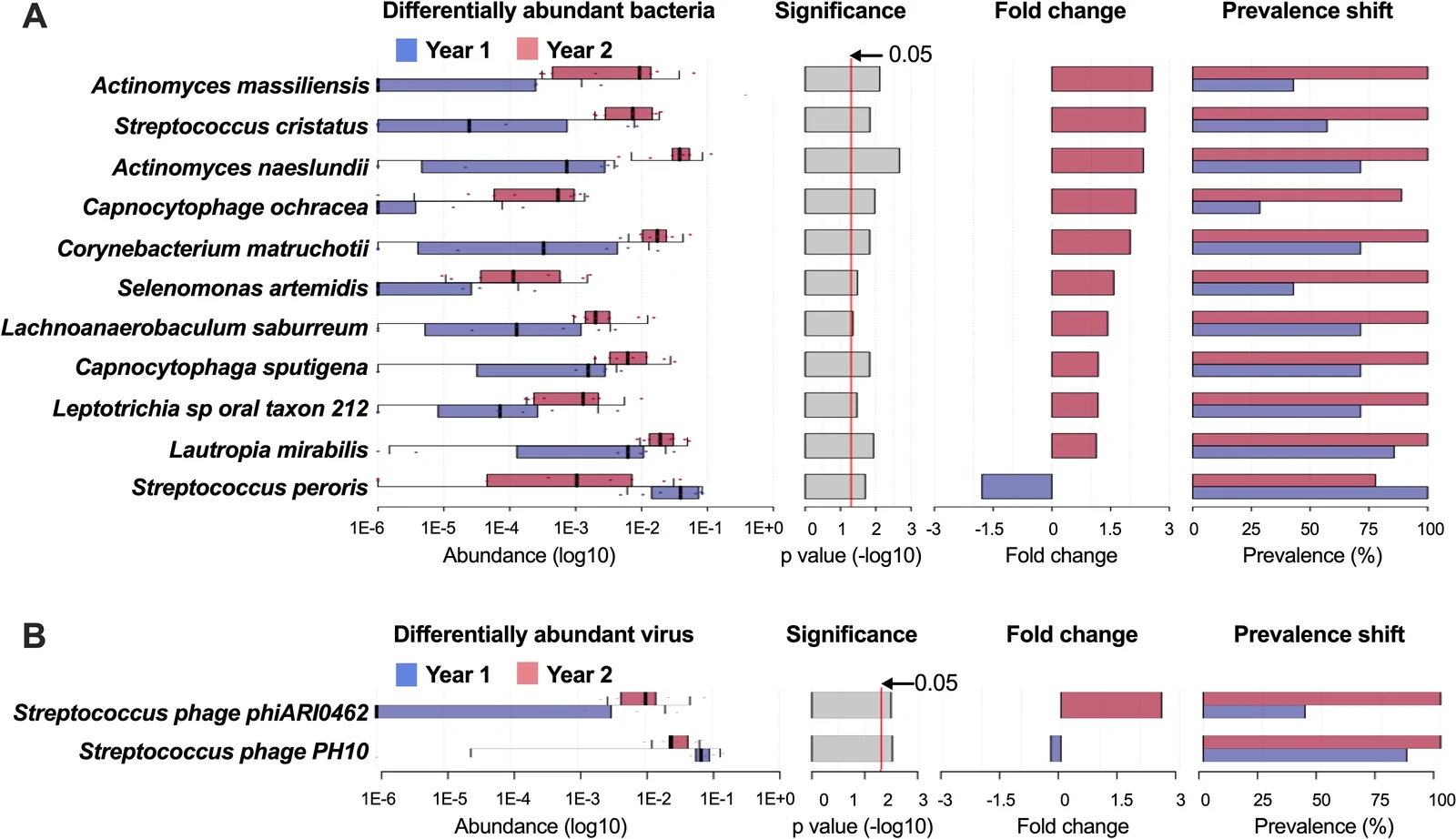
Differentially abundant bacterial and viral taxa between year 1 and year 2. (**A**) Differentially abundant bacterial taxa between year 1 and year 2. (**B**) Differentially abundant viral taxa between year 1 and year 2.

Differential abundance analysis identified age-associated changes across bacterial, viral, and functional pathway profiles. Among 283 bacterial taxa analyzed (Fig. 5A), 11 exhibited significant differential abundance. Of these, 10 were significantly upregulated in year 2: *Actinomyces massiliensis, Actinomyces naeslundii, Streptococcus cristatus, Capnocytophaga ochracea, Corynebacterium matruchotii, Selenomonas artemidis, Lachnoanaerobaculum saburreum, Capnocytophaga sputigena, Leptotrichia* sp. oral taxon 212, and *Lautropia mirabilis. Actinomyces massiliensis* showed the highest log2 fold change, exceeding 2.5, while *Lautropia mirabilis* had a log2 fold change close to 1. Only one bacterial taxon, *Streptococcus peroris,* was significantly downregulated, with a log2 fold change of −1.5.

Among 142 viral taxa (Fig. 5B), two were found to be differentially abundant between year 1 and year 2. *Streptococcus phage phiARI0462* exhibited a log2 fold change of 2.5, indicating a 5.65-fold higher abundance in year 2 compared to year 1. Conversely, *Streptococcus phage PH10* showed a log2 fold change of over −0.4, corresponding to an abundance decrease of more than 25% from year 1 to year 2.

### Functional pathway dynamics and cross-omics integration

**Fig 6:**
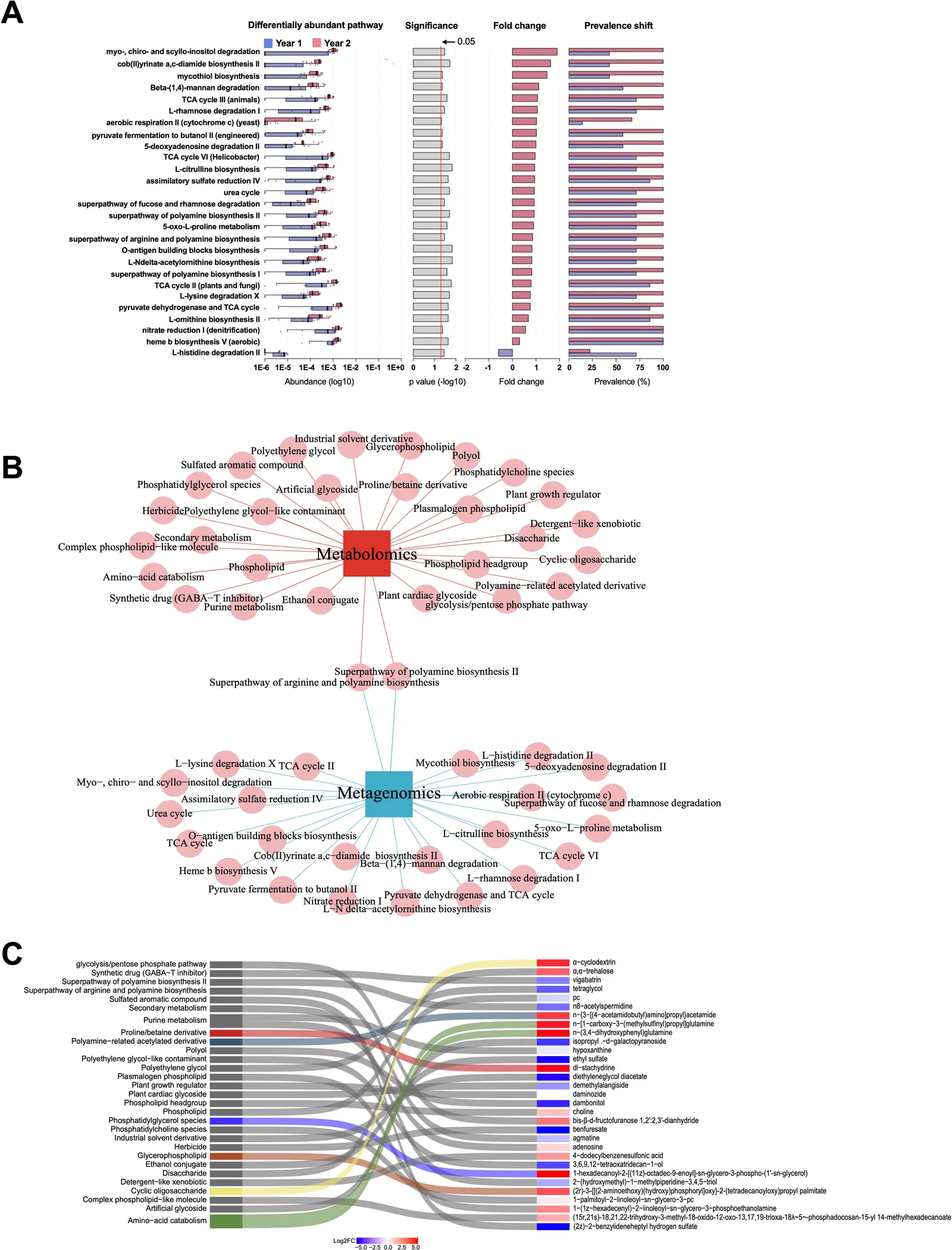
Functional pathway dynamics in infant saliva. (**A**) Differentially abundant functional pathways between year 1 and year 2. (**B**) Functional network linking metagenomic pathways with metabolomic features. (**C**) Sankey diagram illustrating associations between differentially abundant pathways and metabolites.

To focus on biologically relevant changes, differentially abundant pathways were extracted and visualized alongside their associated microbial taxa (Fig. 6A). Of the 28 pathways that differed significantly between year 1 and year 2, 12 exhibited documented links to the salivary microbiome. Notably, the number of pathway-microbiome associations increased at year 2, suggesting tighter functional coupling as the oral ecosystem matured.

As oral bacteria preferentially accumulate within dental plaque, subsequent analyses focused on integrating metagenomic functional profiles with plaque metabolomic pathways. Cross-omics network analysis revealed that only a limited number of pathways served as major connectors between metagenomic functions and metabolomic features (Fig. 6B). In particular, the superpathways of polyamine biosynthesis II and the superpathway of arginine and polyamine biosynthesis emerged as central hubs linking metabolites to functional pathways. The Sankey diagram (Fig. 6C) further showed that *n*8-acetylspermidine and agmatine were the primary differential metabolites associated with these two metabolic pathways.

### Correlation analyses among bacteriome, virome, and metabolites

**Fig 7:**
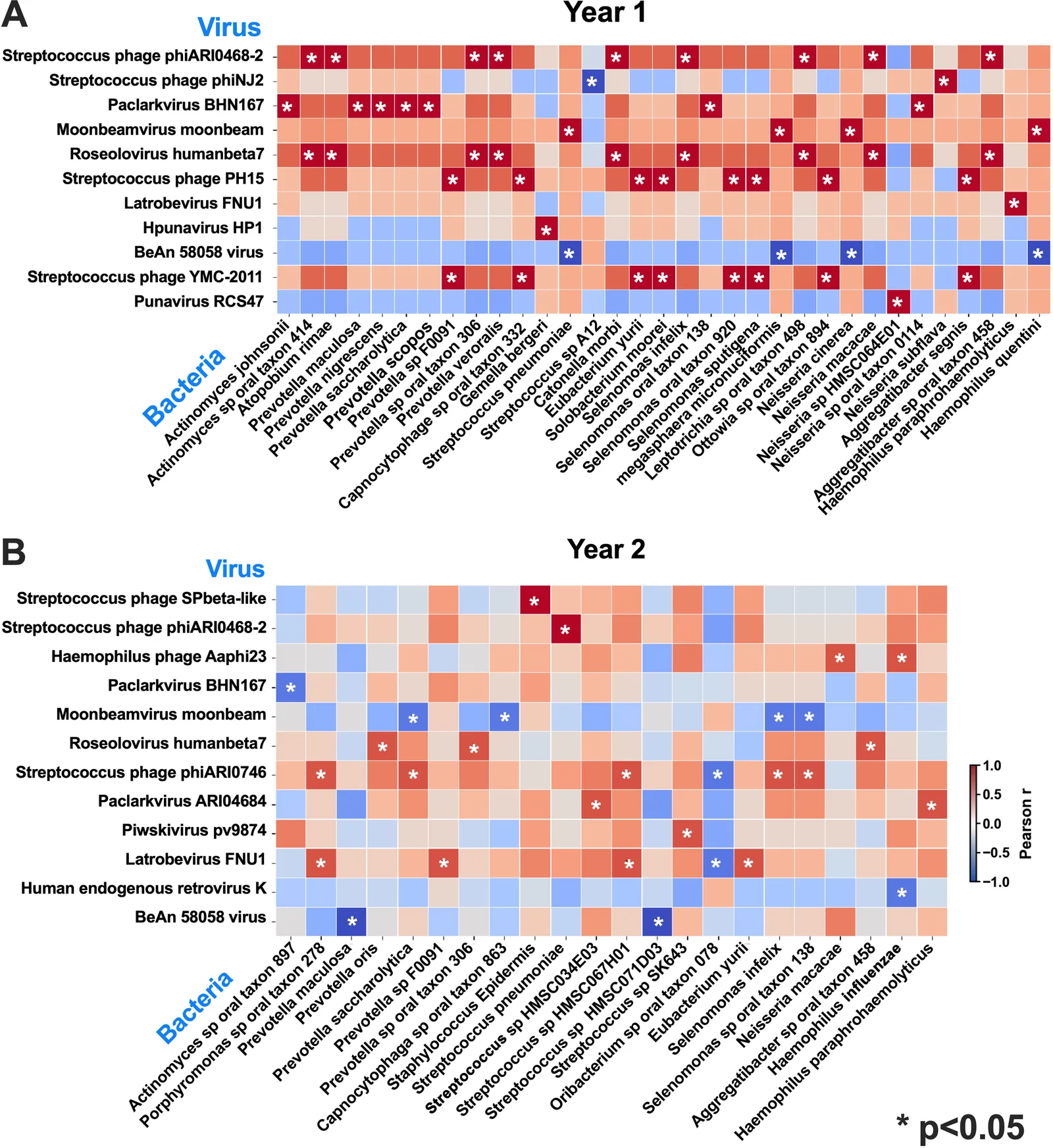
Correlations between the bacteriome and virome. (**A**) Pearson’s correlation analysis between bacterial and viral taxa at year 1. (**B**) Pearson’s correlation analysis between bacterial and viral taxa at year 2.

Overall, correlation strengths between bacterial and viral taxa decreased from year 1 to year 2 (Fig. 7A and B), indicating a progressive reduction in microbial network connectivity. Correlation analysis further revealed distinct patterns of association among microbial taxa and metabolites. Among viral taxa, *Streptococcus* phages, particularly *S*. phage phiARI0468-2 and *S*. phage SPbeta-like, exhibited a higher number of significant correlations with both bacterial taxa and metabolites. Similarly, within the bacterial community, *Streptococcus* species, including *S. pneumoniae*, *S*. sp. A12, and *S. oralis*, consistently showed significant associations with viral taxa and metabolites.

**Fig 8:**
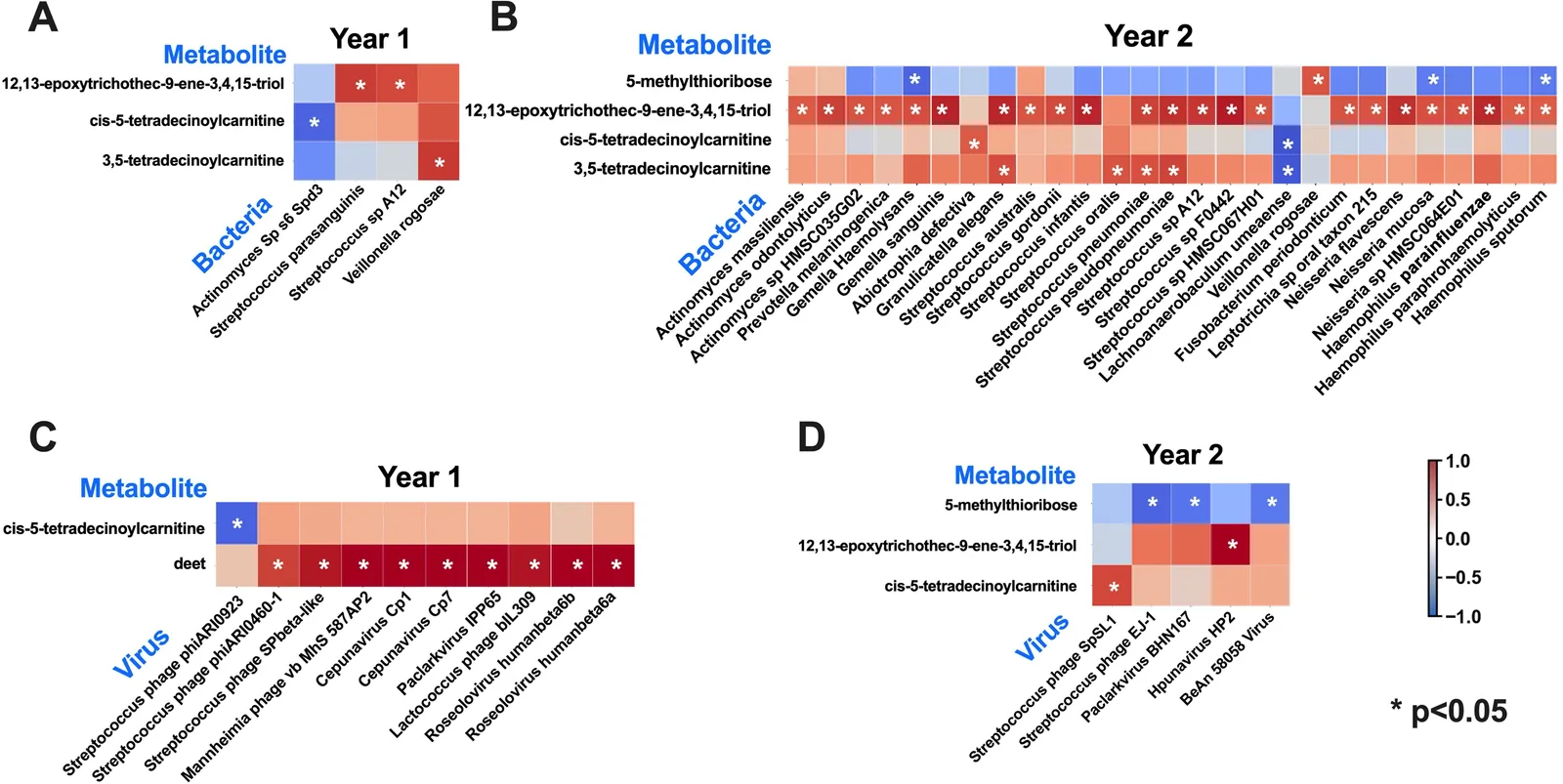
Correlations among the bacteriome, virome, and metabolites. (**A**) Pearson’s correlation analysis between the bacteriome and metabolites at year 1. (**B**) Pearson’s correlation analysis between the bacteriome and metabolites at year 2. (**C**) Pearson correlation analysis between the virome and metabolites at year 1. (**D**) Pearson’s correlation analysis between the virome and metabolites at year 2.

When evaluating correlations with metabolites, bacterial taxa exhibited numerous associations, with a marked increase in correlation density at year 2 compared to year 1 (Fig. 8A and B). In contrast, viral taxa displayed relatively few metabolite associations at either time point (Fig. 8C and D).

## DISCUSSION

This study provides a comprehensive, longitudinal characterization of oral bacteriome, virome, and metabolome development during early life, alongside direct comparisons with maternal metabolomes (10). Untargeted metabolomics first showed that maternal saliva and plaque remained largely stable across visits, whereas infant profiles were clearly distinct from maternal profiles and underwent greater age-related remodeling, particularly in plaque. These findings provided the developmental context for subsequent analysis of paired infant saliva. This study, therefore, presents an integrated, longitudinal assessment of the infant salivary virome in conjunction with bacteriome and metabolome profiles across the first 2 years of life, addressing a critical gap in our understanding of oral viral ecology during early childhood. By applying a multi-omics framework, we reveal temporal dynamics and cross-kingdom associations among bacteria, viruses, and metabolites during a formative period of oral ecosystem maturation. In addition, by profiling both saliva and plaque from mother-infant dyads, we provide paired, site-specific metabolomic insights into oral metabolic development in early life.

### Divergence of infant and maternal metabolomes

Another consistent finding was the relative stability of maternal metabolomes compared with the dynamic remodeling observed in infants. Maternal saliva and plaque showed only minor changes between year 1 and year 2, mainly limited to lipid-related or environmental metabolites. By contrast, infant metabolomes increasingly diverged from maternal profiles, with a growing number of differentially expressed metabolites from year 1 to year 2 in both saliva and plaque. These differences were especially pronounced in pathways related to energy metabolism, amino acid processing, and lipid biosynthesis. Collectively, these results suggest that maternal oral metabolomes remain largely stable, whereas infant metabolomes undergo progressive reprogramming, reflecting intrinsic developmental processes as well as extrinsic influences such as diet, environment, and microbial colonization (11). In this study, maternal metabolomics, therefore, served as an adult reference for interpreting infant-specific development rather than as evidence of direct maternal-infant metabolic transmission.

### Developmental remodeling of the infant oral metabolome

While saliva showed only modest age-related changes, plaque exhibited extensive remodeling from year 1 to year 2, suggesting that plaque is a more dynamic reservoir of metabolic activity (12). In saliva, fungal- and stress-related metabolites decreased with age, whereas environmental xenobiotics such as DEET increased—patterns consistent with reduced microbial stress but greater environmental exposure as infants become more mobile. In plaque, however, declines in xenobiotics and microbial fermentation products were coupled with increases in endogenous metabolites such as polyamines and purine derivatives, reflecting enhanced microbial growth, nucleotide turnover, and biofilm development. The enrichment of plant-derived metabolites in year 2 plaque further highlights the influence of dietary diversification, microbial processing of solid foods, and expanding host-microbe-diet interactions. Taken together, these findings suggest that saliva mainly reflects short-term exposures and stress responses, whereas plaque better captures the long-term trajectory of oral metabolic maturation.

### Metabolic pathway shifts highlight biofilm development and nutrient utilization

Pathway enrichment analyses revealed that infant plaque metabolites were strongly upregulated in central carbohydrate (citrate cycle, glycolysis, and pyruvate metabolism) and amino acid pathways (alanine, aspartate, and glutamate; glycine, serine, and threonine; arginine and proline metabolism). These pathways support the high energy and amino acid turnover required for biofilm expansion (21). One-carbon metabolism and purine metabolism, both critical for nucleotide biosynthesis and methylation, were among the most affected, underscoring their importance in microbial proliferation and biofilm stability. By year 2, lipid-related pathways such as unsaturated fatty acid biosynthesis and fatty acid degradation became enriched, suggesting that lipid remodeling and fatty acid utilization gain prominence as the infant microbiome diversifies.

### Opposing regulation within pathways reflects metabolic complexity

Several pathways contained both up- and downregulated metabolites, underscoring the complexity of oral metabolic regulation. For example, in steroid hormone biosynthesis, cholesterol sulfate decreased consistently in infants, estrone glucuronide increased, and 2-hydroxyestradiol showed age-dependent fluctuations, patterns suggesting dynamic remodeling of estrogen metabolism (22). In purine metabolism, increased sulfate coincided with decreased hypoxanthine and adenosine, reflecting selective nucleotide turnover. Similar mixed trends were observed in glycine/serine/threonine metabolism, fatty acid synthesis, and propanoate metabolism, where different intermediates were differentially regulated. These findings highlight that oral metabolic remodeling is not uniform but instead reflects fine-tuned regulation at specific nodes, likely shaped by interactions among host metabolism, microbial activity, and diet (23). Accordingly, pathway enrichment should not be interpreted as uniform activation of every metabolite within a pathway.

### Coordinated remodeling and decreasing interdependence in the infant oral ecosystem

We observed consistent age-associated changes in both alpha and beta diversity of the salivary bacteriome and functional pathways, indicating substantial compositional and functional restructuring between year 1 and year 2. Cross-omics associations, however, did not change uniformly. Bacteria-virus correlations became less extensive from year 1 to year 2, whereas bacteria-metabolite correlations increased and virus-metabolite associations remained comparatively sparse. Thus, the oral ecosystem appears to undergo network reorganization rather than a generalized loss of connectivity. The weakening of bacteria-virus associations may reflect a developmental transition from an early, highly interdependent colonization network toward a more stable and spatially compartmentalized ecosystem, whereas the increase in bacteria-metabolite associations may indicate that established bacterial communities produce or consume metabolites more consistently as biofilms mature.

Several biological transitions between 1 and 2 years could contribute to these patterns. The introduction of a wider range of solid foods increases carbohydrate, amino acid, lipid, and plant-derived substrates; tooth eruption expands non-shedding surfaces and promotes spatially structured plaque communities; repeated colonization and competition can stabilize core microbial membership; and maturation of mucosal immunity may impose new selective pressures on bacteria and viruses (3, 17, 18, 24). Together, these processes could reduce simple one-to-one bacterial-phage co-occurrence while strengthening the correspondence between established bacterial communities and the metabolites generated or consumed within local oral niches. Because diet, tooth eruption, and immune markers were not modeled directly, these mechanisms remain hypotheses for future testing.

The oral microbiome is a dynamic ecosystem, with core taxa such as *Streptococcus, Veillonella, Rothia, Actinomyces, Gemella, and Prevotella* consistently dominating the bacteriome across diverse populations and time points (25). Longitudinal analyses reveal that environmental factors, including age and oral hygiene practices, exert a greater influence on shifts in microbial communities than host genetics (26). During the transition from year 1 to year 2, age-related changes in taxa abundance are evident, with increases in *Corynebacterium* and *Veillonella* and a decline in *Streptococcus*, paralleling findings that link these genera to developmental stages and brushing frequency (26). The maturation of the oral microbiota is most pronounced in early childhood, with the detection rates of adult-like microbiota rising sharply between 6 and 18 months and stabilizing by 36 months, suggesting a critical window for microbiota establishment (27). Shifts in *Streptococcus* and *Veillonella* abundance are particularly notable as *Streptococcus* typically decreases. At the same time, *Veillonella* increases with poorer oral hygiene and advancing age, reflecting their respective roles in biofilm formation and caries risk (28). The co-occurrence and interaction between *Streptococcus* and *Veillonella* are central to biofilm dynamics, with *Veillonella* species modulating biofilm mass and acidification, thereby influencing oral health outcomes (24). Socioeconomic status, gender, and lifestyle factors such as smoking further modulate oral microbial diversity and composition, underscoring the multifactorial nature of bacteriome shifts over time (29). These age-dependent shifts are, therefore, consistent with ecological succession accompanying tooth eruption and biofilm maturation, although the small cohort precludes attribution to any single exposure.

The oral virome is a highly diverse and dynamic community, with bacteriophages constituting the majority of detected viral taxa (30). Longitudinal metagenomic analyses reveal that oral viral communities are both personalized and temporally persistent, with certain phage genotypes remaining stable across time points (31). The 25 most abundant viral taxa can collectively account for more than 100% of the detected viral abundance due to overlapping host ranges and methodological factors in metagenomic quantification (30). Phages targeting *Streptococcus pneumoniae* maintained consistent abundances between year 1 and year 2, reflecting the stability of some host-phage relationships in the oral ecosystem (32). In contrast, phages infecting *Homo sapiens* (e.g., Roseolovirus humanbeta6b and humanbeta7) and *Escherichia coli* (e.g., cepunavirus Cp1, Cp7, and punavirus P1) showed significant increases in abundance, suggesting dynamic shifts in viral populations that may be linked to changes in host microbial composition or environmental exposures (33). Oral phage-bacterium interaction networks are complex, with cross-infective phages influencing both commensal and pathogenic bacterial populations, thereby shaping microbial community structure (33). The oral virome serves as a reservoir for virulence and antibiotic resistance genes, which can be transferred among bacteria and potentially impact disease progression (34).

### Predominant increases in differentially abundant taxa and pathways

More than 90% of differentially abundant bacterial taxa, viral taxa, and functional pathways were enriched at year 2, suggesting a general expansion and functional diversification of the oral ecosystem with age. This consistent upward trend suggests progressive enrichment of microbial communities and metabolic capacity during early oral development. Although some features exhibited relatively small fold changes, the overall pattern remains biologically meaningful and reflects coordinated ecosystem maturation. Notably, a limited subset of infant oral microbial taxa and functional pathways showed decreased abundance from year 1 to year 2, highlighting the selective nature of ecological succession and underscoring the need for further investigation into the roles of these declining components during early oral development.

The significant upregulation of *Actinomyces massiliensis* and *Actinomyces naeslundii* in year 2 is consistent with their established role as commensal plaque formers and contributors to both oral health and disease (35). *Capnocytophaga ochracea* and *Capnocytophaga sputigena*, also upregulated, are recognized as core members of the healthy oral microbiome and are associated with periodontal health (36). *Corynebacterium matruchotii*, another taxon with increased abundance, is a key architect of dental biofilm structure and is linked to the formation of dental calculus (37).

Analysis of 142 oral viral taxa revealed two *Streptococcus* phages with significant differential abundance between year 1 and year 2. *Streptococcus phage phiARI0462* showed a marked increase in abundance, with a log2 fold change of 2.5, corresponding to a 5.65-fold rise inyear 2. In contrast, *Streptococcus phage PH10* exhibited a log2 fold change of −0.4, indicating a decrease in abundance of more than 25% over the same period. Shifts in phage abundance, such as those observed here, may reflect changes in host bacterial populations, selective pressures from host immunity, or ecological succession within the oral microbiome (32, 38). Oral phages play a critical role in shaping microbial community structure, influencing biofilm formation, bacterial population dynamics, and the transfer of antimicrobial resistance genes (33). Cross-infective phages can modulate the balance between commensal and pathogenic bacteria, with potential implications for oral health and disease progression (33). The observed increase in *Streptococcus phage phiARI0462* and decrease in *PH10* may signal underlying changes in *Streptococcus* host populations or broader shifts in oral microbial ecology (33, 39). Overall, these findings underscore the importance of monitoring phage dynamics in the oral microbiome as the differential abundance of specific phages can serve as indicators of microbial community shifts and inform future strategies for oral health management and phage-based therapeutics (33, 39). The virome exhibited stable alpha diversity but significant changes in beta diversity between year 1 and year 2. This pattern is consistent with the known characteristics of the oral virome, which is highly personalized and temporally persistent at the individual level, yet exhibiting compositional shifts in response to changes in bacterial host populations (31).

### Implication of cross-omics association

The observed correlations highlight biologically meaningful interactions within the developing infant oral ecosystem. The strong associations between *Streptococcus* phages (e.g., phiARI0468-2 and PH15) and their bacterial hosts (*Streptococcus* spp. and *Prevotella* spp.) at year 1 likely reflect active phage-host dynamics that shape bacterial community structure through lytic predation and lysogenic modulation (Fig. 7A and B). Phages shape bacterial communities by killing specific members and also affect the metabolism and virulence of their host species through phage-encoded genes (40). Lytic phages and prophages in saliva, oral mucosa, and dental plaque interact with the oral microbiota and can alter biofilm formation, underscoring their ecological role in the oral cavity (16). In the infant gut, the bacteriophage-bacteria relationship begins from birth with a high predator-low prey dynamic consistent with the Lotka-Volterra model, suggesting that similar predatory dynamics may operate in the developing oral ecosystem (41). The correlations between acylcarnitines (e.g., cis-5-tetradecinoylcarnitine) and multiple *Streptococcus* and *Neisseria* species (Fig. 8A and B) suggest bacterial involvement in fatty acid β-oxidation pathways, potentially reflecting host mucosal metabolic maturation. Acylcarnitines are formed through the esterification of L-carnitine during mitochondrial fatty acid β-oxidation and serve as indirect markers of altered mitochondrial metabolism, while bacteria can transport and metabolize carnitine as a nutrient source or osmoprotectant (42, 43). Similarly, the shift in Roseolovirus (HHV-6/7)-bacteriome correlations from year 1 to year 2 may indicate evolving virus-immune-microbiome interplay during this critical developmental window (44). Primary HHV-6 infection occurs predominantly between 9 and 21 months of age, with salivary glands serving as the main site of viral replication and shedding, aligning temporally with the observed shift in virus-bacteriome associations (45). Phage predation not only directly impacts susceptible bacteria but also leads to cascading effects on other bacterial species via interbacterial interactions, with metabolomic profiling revealing that phage-driven shifts in the microbiome have direct consequences on the gut metabolome (46). Furthermore, bacteriophages have been proposed as tools to modulate the production of microbial metabolites, supporting the notion that these tripartite phage-metabolite-bacteriome associations are functionally interconnected rather than coincidental (47).

### Implications and future perspectives

Overall, our findings highlight the infant oral metabolome as a highly dynamic and adaptive system, progressively diverging from the metabolic stability of mothers as it integrates dietary, microbial, and environmental inputs. Plaque, more than saliva, emerges as a sensitive matrix for capturing these developmental changes, reflecting both microbial metabolism and host-diet interactions. The prominent involvement of one-carbon metabolism, purine metabolism, and lipid pathways underscores their central role in shaping microbial growth and host-microbe symbiosis during early life. Furthermore, the observation of opposing regulatory patterns within the same pathway illustrates the nuanced and multidirectional nature of metabolic remodeling. Future studies integrating metabolomics with microbiome sequencing and dietary assessments and host immune measurements will be essential to link these shifts to specific taxa, exposures, and oral health outcomes (48).

### Study limitation

This study should be considered exploratory and hypothesis-generating. The small sample size (*n* = 10 mother-infant pairs) limits statistical power and generalizability, and findings should be interpreted with caution. Larger, independent cohorts will be required to validate these observations and assess their broader applicability.

In addition, maternal metagenomic sequencing was not performed, which precluded direct comparisons of bacteriome and virome profiles between mothers and infants, including beta-diversity and strain-level analyses. Robust strain-level inference also requires deeper sequencing and specialized analytical approaches; therefore, strain-sharing and transmission could not be reliably assessed in the current data set. Maternal metabolomics was consequently used as a mature adult reference and should not be interpreted as evidence of microbial or metabolic inheritance.

Finally, important factors such as diet, feeding practices, antibiotic exposure, oral hygiene, tooth eruption, and socioeconomic variables were not formally incorporated into multivariable analyses due to the limited sample size, leaving potential residual confounding. This limitation is particularly relevant to plant-associated metabolites and putative environmental exposure features. Untargeted metabolite annotations, including DEET, require targeted confirmation.

The cohort design, while providing a relatively uniform maternal baseline (the mother achieved oral disease-free status before delivery), may also limit generalizability. Future studies with larger cohorts, detailed dietary and exposure records, paired maternal-infant metagenomic sequencing, strain-resolved and phage host-linking analyses, targeted validation of key metabolites, and measurements of host immune development will be essential to investigate microbial transmission, functional relationships, and the relevance of these developmental patterns to early childhood caries and other oral health outcomes.

### Conclusion

Untargeted metabolomics showed that maternal saliva and plaque remained comparatively stable across visits, whereas infant profiles were clearly distinct and underwent stronger longitudinal remodeling, especially in plaque. These findings provided the developmental framework for subsequent analysis of paired infant saliva, which revealed age-related developmental dynamics of the bacteriome, virome, metagenomic functional pathways, and their cross-OMICS associations. Bacteria-virus associations became less extensive, whereas bacteria-metabolite associations were denser when transitioning from age 1 to age 2, indicating OMICS-specific network reorganization. Our study findings provide new insights into the complex and dynamic development of the oral microbiome, virome, and metabolome in early infancy.

## Data Availability

All data generated or analyzed during this study are included in this article. Further inquiries can be directed to the corresponding author. Metagenomic sequencing data have been deposited at Sequence Read Archive (SRA) with the accession number PRJNA1395144 and are accessible at this link: https://www.ncbi.nlm.nih.gov/sra?term=PRJNA1395144&cmd=DetailsSearch. Metabolomics data are deposited to Figshare and accessible at https://doi.org/10.60593/ur.d.31316797.v2. No specific codes were generated different from the published analysis package described in Materials and Methods.

## SUPPLEMENTAL MATERIAL

Supplemental Figures msystems.00946-26-s0001.docx Figures S1-S7.

Data S1 and S2 msystems.00946-26-s0002.xlsx Infant salivary and plaque metabolites.

ASM does not own the copyrights to Supplemental Material that may be linked to, or accessed through, an article. The authors have granted ASM a non-exclusive, world-wide license to publish the Supplemental Material files. Please contact the corresponding author directly for reuse.
