# Supplementary material for: Longitudinal development of infant oral ecosystem: salivary metabolomic, bacteriome, and virome dynamics in early infancy
Source: mSystems. 2026 Aug 31;11(9):e00946-26. doi: 10.1128/msystems.00946-26 (PMC13595985; doi:10.1128/msystems.00946-26)
Supplement: Supplementary file 1 [file msystems.00946-26-s0001.docx]

**Supplemental Figures**

**Fig S1** Plaque metabolomic differences between infants and mothers. (A) PCA scores plot of untargeted metabolomics data for plaque; each dot represents a biological sample. (B) Volcano plot showing differential metabolites in Year 2 versus Year 1 maternal plaque, with 1 upregulated metabolite (p < 0.05, log₂FC < −1). (C) Volcano plot of differential metabolites in Year 1 infant versus Year 1 maternal plaque, showing 190 upregulated (p < 0.05, log₂FC > 1) and 20 downregulated (p < 0.05, log₂FC < −1) metabolites. (D) Volcano plot of differential metabolites in Year 2 infant versus Year 2 maternal plaque, showing 212 upregulated (p < 0.05, log₂FC > 1) and 8 downregulated (p < 0.05, log₂FC < −1) metabolites. (E) Pathway enrichment analysis of up- and downregulated metabolites in Year 1 plaque based on human metabolic pathways. (F) Pathway enrichment analysis of up- and downregulated metabolites in Year 2 plaque based on human metabolic pathways.

**Fig S2** Opposing metabolite regulation in steroid hormone biosynthesis. Pathway enrichment analysis of upregulated metabolites using MetaboAnalyst 6.0 based on human pathway libraries.

**Fig S3** Selective regulation of purine metabolism in infant plaque.

**Fig S4** Glycine, serine, and threonine metabolism remodeling in infant oral samples.

**Fig S5** Divergent regulation of fatty acid synthesis in saliva.

**Fig S6** Opposite trends in propanoate metabolism in infant saliva.

**Fig S7** Remodeling of alanine, aspartate, and glutamate metabolism in infant saliv
